# A novel assay for the detection of bioactive volatiles evaluated by screening of lichen-associated bacteria

**DOI:** 10.3389/fmicb.2015.00398

**Published:** 2015-05-01

**Authors:** Tomislav Cernava, Ines A. Aschenbrenner, Martin Grube, Stefan Liebminger, Gabriele Berg

**Affiliations:** ^1^Institute of Environmental Biotechnology, Graz University of TechnologyGraz, Austria; ^2^Institute of Plant Sciences, University of GrazGraz, Austria; ^3^Roombiotics GmbHGraz, Austria

**Keywords:** volatiles, VOCs, antifungal, antibacterial, lichen symbiosis

## Abstract

Volatile organic compounds (VOCs) produced by microorganisms are known both for their effect on pathogens and their role as mediators in various interactions and communications. Previous studies have demonstrated the importance of VOCs for ecosystem functioning as well as their biotechnological potential, but screening for bioactive volatiles remained difficult. We have developed an efficient testing assay that is based on two multi-well plates, separated by a sealing silicone membrane, two tightening clamps, and variable growth media, or indicators. The experiment design as presented here is a novel and robust technique to identify positive as well as negative VOC effects on the growth of a target organism and to test for specific substances e.g., hydrogen cyanide which can be detected with a suitable indicator. While the first pre-screening assay is primarily based on indicator color change and visible growth diameter reduction, we also introduce an advanced and quantitatively precise experiment design. This adaptation involves qPCR-based quantification of viable target cells after concluding the treatment with VOCs. Therefore, we chose preselected active isolates and compared the partial 16S rRNA gene copy number of headspace-exposed *E. coli* with non-treated controls. Separately obtained headspace SPME and GC/MS-based profiles of selected bacterial isolates revealed the presence of specific and unique signatures which suggests divergent modes of action. The assay was evaluated by screening 100 isolates of lung lichen-associated bacteria. Approximately one quarter of the isolates showed VOC-based antibacterial and/or antifungal activity; mainly *Pseudomonas* and *Stenotrophomonas* species were identified as producers of bioactive volatiles.

## Introduction

Volatile organic compounds (VOCs) are organic compounds that have a high vapor pressure at ordinary room temperature. VOCs are produced by the majority of organisms and they often function as communication molecules (Effmert et al., 2012). The most notable characteristic of all VOCs is the extent of their range of influence as compared to non-volatile substances. While other secreted metabolites rely on close contact between interacting organisms or diffusion through separating matter, VOCs can overcome much greater distances. Bacterial as well as fungal strains produce a broad spectrum of bioactive VOCs with multi-functional effects, which are not restricted to the same species. Their action across organismal kingdoms was shown by Ryu et al. (2003) who demonstrated that bacterial volatiles promoted growth in *Arabidopsis thaliana*. In contrast, some bacterial isolates were also shown to reduce the growth of *A. thaliana* through emission of bioactive volatiles (Vespermann et al., 2007; Blom et al., 2011; Weise et al., 2013). Moreover, bacterial VOCs were shown to be able to suppress the growth of soil-borne pathogenic fungi, e.g., *Rhizoctonia solani* (Kai et al., 2007). Bacteria are able to communicate over long distances within the root system, both among bacteria and with plant hosts, where they elicit induced systemic resistance (ISR) and growth promotion (Farag et al., 2013). VOCs emitted by different soil bacteria can affect the growth, antibiotic production, and gene expression of soil bacteria (Garbeva et al., 2014). Owing to these multi-functional roles of VOCs, they have an enormous potential for biotechnological applications (Strobel, 2006; Schalchli et al., 2014). Currently, there is no efficient testing assay that allows for rapid screening of bioactive volatile metabolites in interrelation between two different microorganisms within the same headspace.

Host-associated microbiomes are important reservoirs for VOC-producing organisms because communication and pathogen defense are essential functions of the microbiome, and recently shown to be integral for healthy plant and human life (Blaser et al., 2013; Philippot et al., 2013). For the rhizosphere microbiome located on/in plant roots, the proportion of VOC producers is often high because in plants the root-associated microbiome acts as a primary protection shield against soil-borne pathogens (Cook, 1990; Weller et al., 2002). A similar role was also attributed to bacteria in the self-sustaining lichen symbiosis (Grube et al., 2015). In each microbiome a certain proportion of microorganisms with antagonistic activity against pathogens is involved in this function. Using a combination of metagenomic, -proteomic, and cultivation approaches, a proportion of 7% antagonists was identified for the lung lichen (Grube et al., 2015). Identification of antagonistic microorganisms is still a challenge (Berg et al., 2013), but nevertheless important for a more profound understanding of ecosystem functioning and also a necessary tool for bioprospecting in biotechnology (Strobel, 2006). The discovery of novel bioactive compounds facilitates improvement in disinfection strategies and drug discovery, both of which are in high demand due to the increasing rates of resistance to antibiotics (Woolhouse and Farrar, 2014). Antagonistic microorganisms harbor a vast potential to produce active biomolecules for direct activity against pathogens but also for mediators in various interactions, e.g., pathogen defense, quorum sensing, microorganism-host-interaction. Some of these biomolecules are highly active modifications of known antimicrobial substances and are therefore less susceptible to existing resistance mechanisms. In the past, most efforts focused on antibiotics for which high-throughput screening strategies were already developed (Conery et al., 2014; Seyedsayamdost, 2014). Although previous studies have demonstrated promising effects of bacterial and fungal volatile compounds, they are difficult to detect as well as to identify. Due to their inspiring odors, lichen extracts are used as raw materials in perfumery (Joulain and Tabacchi, 2009). As the specificity of bacterial communities in this phylogenetically old symbiosis was only recently detected (Grube et al., 2009), nothing is yet known about VOCs produced by the abundant lichen-associated bacteria.

**Figure 1:**
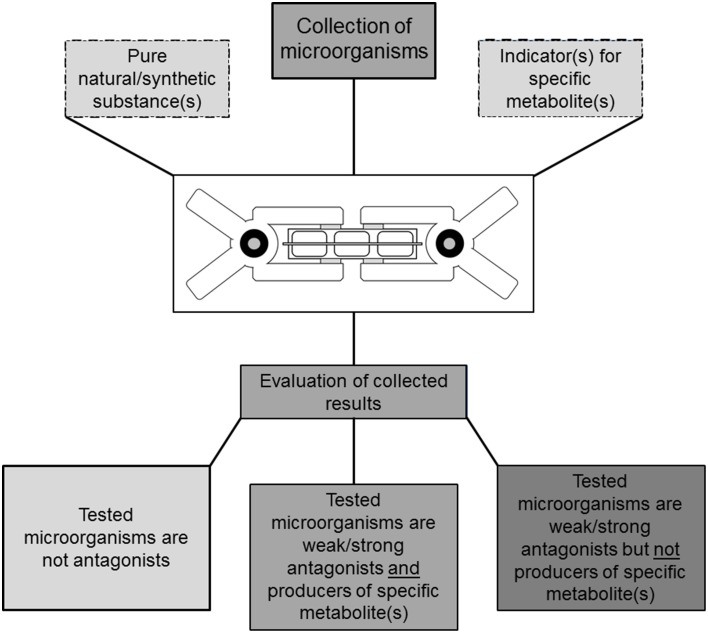
**Illustration of a TCVA-based screening approach for bioactive volatile compounds**. The experiments are based on microorganism cultures suitable for cultivation in well-plates. Parallel setup of different experiments facilitates selectivity and can be used to eliminate producers of predefined compounds e.g., HCN if indicator substances are available. Utilization of additional reference substances enables gradual evaluation of visually inspected experiments.

The objective of our study was to develop a well plate-based and cost-effective testing assay for the emission of bioactive VOCs. We chose lichen-associated bacteria for evaluation purposes. One hundred lichen-associated bacterial isolates were tested for volatile antagonistic activity in order to evaluate our assay. A noteworthy screening assay for biological hydrogen production developed by Schrader et al. (2008) was used as the basis for developing our testing system. The assay is based on two micro-well plates, separated by a sealing silicone membrane, two tightening clamps, and variable growth media or indicators. The suggested experiment design can be used to differentiate between target organism inhibition or growth promotion by a pure substance and also for the same effects caused by volatile mixtures emitted by living microorganisms. This, as well as an increased throughput compared to classic single plate-tests illustrates the novelty of the presented assay in comparison to already described experiment setups. In addition, it can be employed to test for specific substances which can be detected with a suitable indicator (Figure 1). Using this design we identified 30 out of 100 lichen-associated bacterial isolates, which produced bioactive volatiles and induced growth inhibition in two distinct target organisms. Since many lichenicolous organisms are characterized by slow growth rates and difficult or impossible to grow on media, two classic model targets were employed for evaluation purposes. *E. coli* was used in this experimental approach as a model for a typical human pathogen and *B. cinerea* as a model for a plant pathogen. Additional GC/MS-based headspace analysis with different lichen-associated isolates was applied to demonstrate the occurrence of isolate-specific VOC profiles.

## Material and methods

### Isolation of lichen-associated bacteria

*Lobaria pulmonaria* lichen thalli were sampled from three different locations in Austria (Tamischbachgraben, N 47°32′40″, E 14°37′35″, Johnsbach, N 47°38′07″, E 14°44′45″, and St. Oswald ob Eibiswald, N 46°44′50″, E 15° 04′26″). The lichen samples were ground with mortar and pestle, and subsequently combined with a ratio of 1:10 0.85% sterile NaCl in a lab stomacher to form a homogenate (BagMixer; Interscience, St Nom, France). The diluted fractions were then plated onto agars R2A (Carl Roth, Karlsruhe, Germany), R2A with 25 μg ml^−1^ cycloheximide, starch casein agar (SCA; Küster and Williams, 1964), and ISP2 (Shirling and Gottlieb, 1966). Distinctive bacterial colonies were transferred onto R2A plates for sub-cultivation within 5 days of incubation at room temperature. After subsequent testing for antagonism against different pathogens among other physiological tests, 100 lichen-associated bacterial isolates were selected out of 388 available isolates from the in-house culture collection. All of these isolates met at least one of the following criteria: (i) antagonistic activity against *E. coli* K12, (ii) antagonistic activity against *Staphylococcus aureus* ATCC 25923, (iii) antagonistic activity against *Botrytis cinerea* (SCAM, culture collection of the institute of Environmental Biotechnology, Austria), (iv) antagonistic activity against *Rhinocladiella* sp. (culture collection of the Institute of Plant Sciences, University of Graz) in dual-culture experiments, (v) chitinase activity on chitin agar and in chitin-RBV assay, (vi) β-glucanase activity with chromogenic AZCL-Barley β-glucan.

### Two clamp VOCs assays (TCVAs)

Depending on the experiment type, 6-, 12-, and 24-well plates (Greiner Bio-One, Frickenhausen, Germany) were used together with a perforated (0.5 cm ø) 1 mm silicone foil (detailed specifications are presented in Table S1) for tightening connected wells and usual clamps for fixation. Sterile plates were acquired and the silicone foils used were washed and autoclaved at 121°C (holding time of 20 min). 6-, 12-, and 24-well plates were filled with respectively 5, 3.5, and 1.5 mL sterile media per well. The preparation steps and the final setup are pictured sequentially in Figure S1.

### TCVA with *B. cinerea* and lichen-associated bacteria

**Figure 2:**
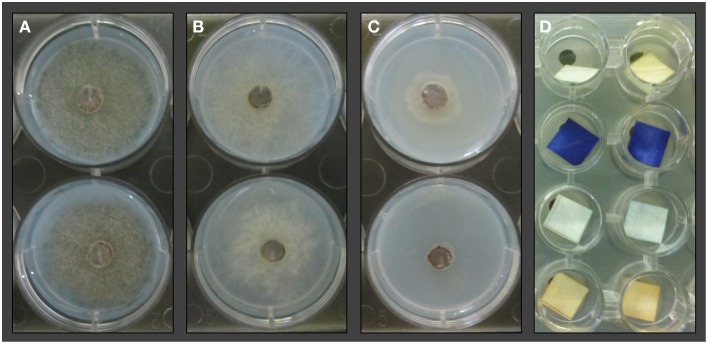
**TCVA with lichen-associated bacterial isolates and *Botrytis cinerea***. Mycelium growth and sporulation was compared to untreated controls **(A)** after 4 days of co-incubation. Inhibition of sporulation was recorded for wells where discoloring of the mycelium was not observable **(B)**. Inhibition of mycelial growth was recorded for wells with 50% or less mycelium proliferation **(C)** compared to negative controls. TCVA with HCN indicator **(D)** based on copper(II) ethyloacetoacetate and 4,4'-methylenebis(N,N-dimethylaniline). The second row shows positive reactions where bacterial isolates from counterpart wells secreted HCN into the headspace which led to the color change of indicator strips.

The bacterial isolates were streaked onto Nutrient Agar (NA; Sifin, Berlin, Germany) in 6-well plates and pre-incubated for 24 h at 30°C. Next, 5 mm diameter plugs were cut from a donor plate evenly covered with *B. cinerea*. These plugs were placed in the center of each well in the 6-well plates containing Synthetic Nutrient-Poor Agar (SNA). After the inoculated plates were checked for sufficient growth, silicone foils were placed between plate pairs containing lichen isolates and *B. cinerea*, respectively. The plates were then clamped together; the lichen-associated bacteria plate was placed upside-down over the *B. cinerea* plates on the bottom. The plates were incubated in the dark at 21°C for 4 days and subsequently visually inspected for mycelium growth and compared to untreated controls (Figure 2A). Two types of controls were implemented; one containing NA wells without any bacteria and one inoculated with *E. coli* K12 instead of lichen-associated isolates.

### TCVA with *E. coli* and lichen-associated bacteria

The bacterial isolates were streaked onto NA in 12-well plates and pre-incubated for 24 h at 30°C. Following the incubation time, a fluid Nutrient Broth (NB; Sifin, Berlin, Germany) culture of *E. coli* K12 was grown to an OD_600_ = 0.4–0.6. 6 mL aliquots were then sequentially transferred to 200 mL NA (20%) supplemented with 0.2 mg/mL 2–(4–iodophenyl)–3–(4–nitrophenyl)–5–phenyltetrazolium chloride (INT; Sigma-Aldrich, St. Louis, MO, USA) and immediately pipetted into sterile 12-well plates. INT can be utilized to detect dehydrogenase activity due to visible color changes. Hence, approximate differences in bacterial abundance can be correlated to the grade of visible discoloring of INT-supplemented growth medium. After solidification of the *E. coli* K12 containing plates, silicone foils were placed between plate pairs containing lichen isolates and *E. coli* K12, respectively. The plates were then clamped together; the lichen-associated bacteria plate was placed upside-down over the *E. coli* K12 plates on the bottom. After 24 h incubation at 21°C, the plates were checked for differences in indicator color change and compared to positive and negative controls. Positive controls were obtained using different commercial disinfectants to determine sufficient OD_600_ values (0.4–0.6) for *E. coli* K12 and an adequate concentration of INT (Figure S2). Two types of negative controls were implemented: one containing NA wells without any bacteria, and one inoculated with *E. coli* K12 instead of lichen-associated isolates.

### qPCR validation of TCVA results

This experiment is an adaption of the aforementioned TCVA with *E. coli* K12, the only modification being that semi-solid 0.3% NA was used instead of solid 1.5% NA in the initial steps of the experiment. After the incubation time, 500 μL of the semi-solid medium with *E. coli* K12 and INT was transferred into 2 mL reaction tubes with 1 mL 0.85% NaCl and subsequently dissolved via vortex. Each tube was supplemented with 10 μL (1:100 solution) propidium monoazide (PMA; GenIUL, Barcelona, Spain) and incubated on ice in the dark while shaking at 100 rpm for 50 min. The tube lids were then opened after incubation and placed under a LED light source for activation of PMA with an emission maximum of 520 nm for 10 min. PMA forms covalent bonds with available DNA but cannot pass through undisrupted cell membranes. This step was performed to exclusively detect gene fragments from living *E. coli* K12 in the qPCR-based quantification. The suspension was then transferred to glass bead containing tubes and mechanically disrupted for 2 × 45 s at 6 m/s with a FastPrep®-24 Instrument (MP Biomedicals Europe, Illkirch, France) and centrifuged at 3000 × g for 2 min to sediment beads and residual Agar. DNA was subsequently extracted from 500 μL of supernatant using the GeneJET Genomic DNA Purification Kit (Thermo Scientific, Waltham, MA, USA). Quantification of 16S rDNA fragments from the DNA extract was conducted with primer pair Unibac-II-515f/Unibac-II-927r as described by Lieber et al. (2003), and standards containing the Unibac-II fragments were prepared according to Köberl et al. (2011). For standard preparation, the gene fragments from *Bacillus subtilis* subsp. *subtilis* Sd3-12 were cloned into the pGEM®-T Easy Vector (Promega, Madison, WI, USA) and later re-amplified with vector specific primers. Total DNA extract treated with amplification-grade DNase I (Sigma-Aldrich, St. Louis, MO, USA) was used to determine the inhibitory effects of co-extracted substances. Based on these results, the extracted DNA was then diluted 1:10 and the target regions were amplified using KAPA SYBR FAST qPCR Kit (Kapa Biosystems, Woburn, MA, USA). Two independent runs with three replicates for each sample were performed on the Rotor Gene 6000 (Corbett Research, Mortlake, Australia) according to Bragina et al. (2013). The specificity of the amplicons and qPCR products was confirmed using melting-curve analysis and gel-electrophoresis, respectively.

### TCVA with a hydrogen cyanide (HCN) indicator and lichen-associated bacteria

The bacterial isolates were streaked onto NA in 24-well plates and pre-incubated for 24 h at 30°C. Indicator strips were prepared using blotting paper submerged in 10 mL chloroform (Carl Roth, Karlsruhe, Germany) solution with 50 mg copper(II) ethylacetoacetate (Sigma-Aldrich, St. Louis, MO, USA) and 50 mg 4,4-methylenebis(N,N-dimethylaniline) (Sigma-Aldrich, St. Louis, MO, USA) and left to air dry. After the pre-incubation time, 1 × 1 cm HCN indicator strips were placed in each well of a 24-well plate. Silicone foils were placed between the upside-down lichen-associated bacteria plates and those containing the HCN indicators. The plate pairs were then clamped together and incubated for 48 h at 30°C. Lastly, the indicator plates were checked for an intense blue color change in the corresponding upper wells. Negative controls were conducted with non-inoculated NA wells.

### Identification of active isolates by 16S rDNA sequencing

Isolated DNA from pure cultures was amplified with primer pair 27F/1492r according to Lane (1991). The PCR product was purified with Wizard® SV Gel and PCR Clean-Up System (Promega, Madison; WI, USA) followed by Sanger sequencing (LGC Genomics, Berlin, Germany). The sequences were aligned with BLASTn (http://blast.ncbi.nlm.nih.gov/Blast.cgi) and 16S ribosomal RNA sequences database. Identification of the closest match was applied for the retrieved results.

### Headspace SPME and GC/MS analysis of bacterial VOCs

The utilized GC/MS SPME headspace method was adapted with minor changes from Verginer et al. (2010). For sample preparation from bacterial isolates, single colonies were transferred with an inoculating loop on 10 mL NA slope agar (1.5%) in 20 mL headspace vials (75.5 × 22.5 mm; Chromtech, Idstein, Germany). The isolates were streaked out in 3 parallel lanes to ensure similar bacterial lawn density after incubation. Following 48 h of incubation at 30°C the vials were sealed with adequate crimp seals and incubated for additional 2 h. Solid phase micro extraction (SPME) was performed with an automated sampler and 50/30 μm Divinylbenzen/CarboxenTM/ Polydimethylsiloxane (PDMS) 2 cm Stableflex/SS fiber (Supelco, Bellefonte, PA, USA). Volatile compounds were enriched for 30 min at 30°C. Compound separation and detection was performed on a system combining a GC 7890A with a quadrupol MS 5975C (Agilent Technologies, Waldbronn, Germany). Samples were run through a (5%-phenyl)methylpolysiloxane column, 60 m × 0.25 mm i.d., 0.25 μm film thickness (DB-5MS; Agilent Technologies, Waldbronn, Germany), followed by electron ionization (EI; 70 eV) and detection (mass range 25–350). The inlet temperature was adjusted to 270°C. For the temperature gradient the GC column was kept at 40°C for 2 min, raised to 110°C at a rate of 5°C/min, then to 280°C at 10°C/min and finally maintained at 280°C for 3 min. The helium flow rate was set to 1.2 mL/min. Serial analysis was done with up to 12 samples per run. Obtained spectra were compared with NIST Mass Spectral Database 08 entries. Specific compounds were identified based on their retention indices and comparison to reference substances (Sigma-Aldrich, St. Louis, MO, USA). Origin 8.5 (OriginLab, Northampton, MA, USA) was applied for visualization of total ion chromatograms (TICs). Background-subtracted mass spectra were used for the depiction of unidentified substances.

### Statistical analysis

The statistical analysis was conducted with ANOVA within RStudio (version 0.97.551) and one-sided *t*-test (*P* < 0.001). Gene copy numbers of the UniBac-II fragment from TCVA-exposed samples (*n* = 36) were compared to untreated controls (*n* = 12). The gene copy numbers were obtained from two biological samples and three qPCR repeats respectively.

## Results

### Testing volatile activity against *E. coli* and botrytis cinerea

The here presented Two Clamp VOCs Assay (TCVA) made it possible to detect bioactive VOC producers within 100 lichen-associated bacterial isolates. Sporulation reduction (Figure 2B) was demonstrated for five isolates; four isolates reduced sporulation of *B. cinerea* in three out of four replicate experiments, while one isolate reduced sporulation in all four trials. *B. cinerea* growth was repeatedly reduced after exposure to 21 different lichen-associated bacterial isolates in the TCVA, and mycelium proliferation was visibly affected (Figure 2C) for these isolates when compared to negative controls. Moreover, 16 isolates reduced proliferation in three out of four replicate experiments, while five isolates reduced proliferation in all four trials. Only one of the identified growth-reducing isolates was later shown to release HCN into the headspace. TCVAs with *E. coli* allowed identification of 10 lichen-associated isolates that are associated with the exertion of antagonistic activity through headspace. Low INT-based growth media discoloring indicating a reduced number of metabolically active bacteria was observed in all three replicate experiments. Comparison to the corresponding HCN TCVAs showed that two of the growth-reducing isolates did not release HCN into headspace. Only one isolate inhibited the growth of both target organisms and was later identified as *Pseudomonas umsongensis* 313P5BS. From all identified antagonists we have selected the 15 most active isolates against one or both target organisms and one non-inhibiting isolate for Sanger sequencing (Table 1).

**Table 1:** **Overview of identified isolates including corresponding activity in TCVAs**.

**Strain ID**	**Closest BLASTn match**	**GenBank accession #**	**Inhibition of *E. coli***	**Inhibition of *B. cinerea***	**HCN producer**
43P2BR	*Bacillus pumilus*	KP739785		✓	
236P5S	*Pseudomonas umsongensis*	KP739786	✓		✓
268P3S	*Pseudomonas umsongensis*	KP739787	✓		✓
269P3R	*Burkholderia sordidicola*	KP739788	✓		
271P3S	*Pseudomonas umsongensis*	KP739789	✓		✓
279P5I	*Pseudomonas umsongensis*	KP739790	✓		✓
288P4R	*Burkholderia sordidicola*	KP739791	✓		
293P5BI	*Pseudomonas umsongensis*	KP739792	✓		✓
300P5BR	*Chryseobacterium piscium*	KP739793		✓	
301P5BS	*Pseudomonas umsongensis*	KP739794	✓		✓
313P5BS	*Pseudomonas umsongensis*	KP739795	✓	✓	✓
409P5	*Pseudomonas lini*	KP739796	✓		✓
418P4B	*Stenotrophomonas rhizophila*	KP739797		✓	
439P1B	*Stenotrophomonas rhizophila*	KP739798		✓	
460P5B	*Stenotrophomonas rhizophila*	KP739799		✓	
471P3B	*Bacillus pumilus*	KP739800			✓

*Listed species represent the closest match of BLASTn searches within the 16S ribosomal RNA sequences database (NCBI). The 16S gene fragment sequences were deposited at GenBank (http://www.ncbi.nlm.nih.gov/genbank)*.

### Screening for HCN producers

All lichen isolates were tested for HCN production in a modified TCVA by imposing indicator strips to the headspace. Nine bacterial isolates induced dark blue discoloring of the indicator strips in all three replicate experiments. Eight of the identified HCN-producing isolates also reduced growth of *E. coli* in previous experiments. These isolates were later assigned to *Pseudomonas* spp., while the non-antagonistic HCN producer had the highest sequence similarity to a *Bacillus pumilus* isolate.

### Validation of TCVA results by quantitative PCR

**Figure 3:**
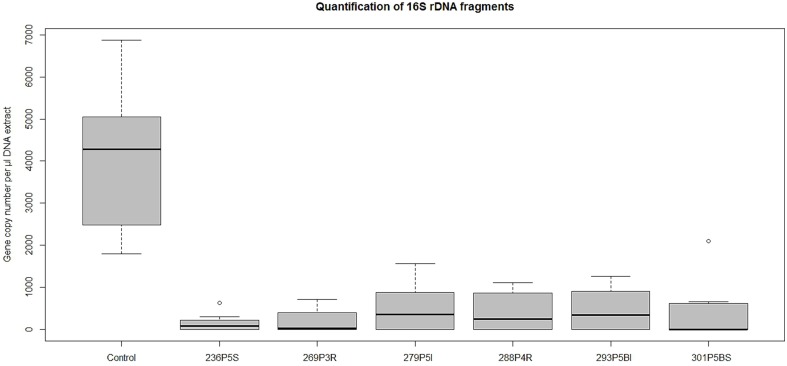
**A qPCR approach was used to determine gene copy numbers of *E. coli* K12 after exposition to strong VOCs antagonists**. TCVA-exposed samples were treated with PMA and gene copy numbers of viable cells were quantified with Unibac-II primers. Values for the treated samples were obtained from respectively six qPCR runs and additional 12 qPCR runs for untreated controls. Statistical analysis with ANOVA and one-sided *t*-test confirmed a highly significant decrease of gene copy numbers of the treated samples compared to the control group (*P* < 0.001).

DNA extracts from wells containing *E. coli* that had shown low discoloring of INT after exposition to lichen-associated bacteria were used to determine the gene copy number of the Unibac-II fragment. DNA from dead or disrupted cells was blocked by PMA which enabled a correlation between gene copy number and living cells. A significantly lower gene copy number compared to controls was shown for all samples exposed to the headspace of highly active antagonists that were pre-screened based on INT discoloring. An approximately 4-fold decrease of the gene copy number was observed with the least inhibiting antagonist *Pseudomonas* sp. 279P5I, while the most effective antagonist *Pseudomonas* sp. 236P5S decreased the gene copy number of *E. coli* approx. 15-fold (Figure 3).

### Taxonomic assignment of active lichen-associated isolates

Sanger sequencing revealed multiple occurrence of some dominant genera. Furthermore, sequencing of 16S rRNA gene fragments from the 15 most active isolates and a non-antagonistic HCN producer revealed the majority belonging to the genus *Pseudomonas* (8 isolates), followed by *Stenotrophomonas* (3 isolates) and three other genera with lower occurrence: *Bacillus, Burkholderia*, and *Chryseobacterium*. Utilization of the TCVA demonstrated that *E. coli* inhibition was mostly observed after exposure to the headspace of *Pseudomonas* sp., while *B. cinerea* growth reduction was mostly observed after exposition to the headspace of *Stenotrophomonas rhizophilia*. Moreover the sequencing approach revealed that different *Pseudomonas* sp. inhibited *E. coli* growth accompanied by HCN release into headspace. Identified isolates are presented together with corresponding TCVA results in Table 1.

### GC/MS-based headspace analysis with selected isolates

**Figure 4:**
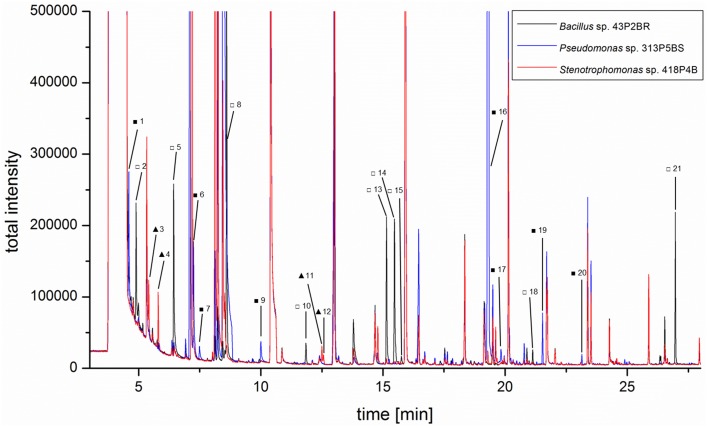
**Three lichen-associated bacterial isolates were subjected to headspace SPME GC/MS analysis to identify isolate-specific VOC profiles**. An overlay of the respective TIC chromatograms illustrates the presence of specific metabolites in the headspace of *Bacillus pumilus*43P2BR (empty squares), *Pseudomonas umsongensis* 313P5BS (filled squares), and *Stenotrophomonas rhizophila* 418P4B (filled triangles). Identified *Bacillus*-specific compounds were: 1-butanol (5□) and 3-methyl-2-pentanone (8□). Methyl thiocyanate (7■) was identified as *Pseudomonas*-specific compound. *Stenotrophomonas*-specific compounds were: 2-butanol (3▴) and 2-methyl-1-propanol (4▴). More details in Table S2.

Three representative isolates which were shown to inhibit growth of headspace-exposed target microorganisms and which were taxonomically assigned to reoccurring genera were used for subsequent GC/MS headspace SPME profiling. Isolate-specific VOCs were identified by overlays of total ion chromatograms (TIC; Figure 4). A total of 21 compounds (Table S2) were found to be unique and only present in TICs of a specific isolate. *Bacillus pumilus* 43P2BR emitted nine distinctive volatile compounds (compound IDs: 2, 5, 8, 10, 13, 14, 15, 18, and 21), followed by *Pseudomonas umsogensis* 313P5BS with eight distinctive compounds (compound IDs: 1, 6, 7, 9, 16, 17, 19, and 20). *S. rhizophila* 418P4B was shown to emit only four distinctive compounds (compound IDs: 3, 4, 11, and 12). Compound identification indicated that *B. pumilus* 43P2BR emitted 1-butanol, 3-methyl-2-pentanone and seven unidentified substances (Figures S3-18). 2-butanol, 2-methyl-1-propanol and two unidentified substances were found within spectra of *S. rhizophila* 418P4B. Conversely, *P. umsogensis* 313P5BS emitted methyl thiocyanate as well as seven unidentified substances.

## Discussion

The results of the screening for bacteria-derived bioactive VOCs demonstrated the applicability of a novel testing system, which is not restricted to bacteria associated with lichens, but can be widely applied with microorganisms sampled in other habitats. With the presented setup several 100 isolates can be tested simultaneously for VOCs-driven effects on target microorganisms and occurrence of specific metabolites. This facilitates screening programs for strain-specific biological effects. Thus, the method is also not limited to observations of growth inhibition such as demonstrated in the present study, but could also be used to identify growth promoting effects. The method, however, reveals the effect of the total “volatilome” of a bacterial strain and does not dissect the effect of individual substances. The composition of the mixture of volatile substances must still be assessed by chemical analysis, after which individual compounds might be tested separately.

We have selected 100 isolates for evaluation purposes that met predefined criteria such as antagonism in classic dual-culture experiments. Thus, we expected to identify a sufficient number of isolates which emit bioactive VOCs to validate the experimental design and the presented workflow. Utilization of different TCVA variations allowed the identification of 30 growth inhibiting bacterial isolates with a novel and reliable technique. Subsequent analysis of the headspace from taxonomically dissimilar bacterial isolates by employing headspace SPME GC/MS demonstrated the presence of isolate-specific TIC profiles and unique compounds in each sample. We have identified five out of 21 unique compounds, emitted by three distinct bacterial strains, to exemplify the presented workflow. While some compounds most likely originate from bacterial degradation of growth media (waste products of bacterial metabolism) and therefore do not target pathogens specifically, other compounds might either be involved in molecular signaling or inhibition of competing organisms. These differences and their significance in natural systems merit further exploration. It might be hypothesized that bacterial bioconversion of the natural substrate may result in volatile compounds with signaling effect. Specifically, the odor of lichens, which may attract reindeer or is part of perfumes, could be influenced not only by the genuine compounds produced by the fungal or algal symbiont, but possibly also by VOCs produced by the bacteria themselves or by compounds released from the fungal matrix due to the metabolic activity of associated bacteria.

The presented workflow includes a pre-incubation of the tested isolates to minimize their inhibition by volatiles emitted by the target organisms. Due to intended growth advantage of the tested organisms, emitted volatiles from the target organisms might not play an important role during the co-incubation. Still, such effects cannot be completely avoided with the presented setup. An inverted approach where the “target” is pre-incubated and subsequently tested against the respective isolate collection could be implemented to obtain a more holistic view on occurring interactions.

Interestingly, *P. umsongensis*, a bacterial species isolated from soil as well as from fungal hyphae and described as “fungiphilic” (Warmink et al., 2009), was the only representative of *Pseudomonas* isolates that consistently inhibited *B. cinerea* growth. This may have resulted from better and faster growth on solid medium compared to the other utilized strains and therefore a higher accumulation of cyanide in the headspace. Various *Pseudomonas* species are known to be cyanogenic bacteria and therefore enhanced toxicity toward various prokaryotes and eukaryotes can be expected even if they are not in close contact. While the employed headspace SPME GC/MS method was not suitable for detection of hydrogen cyanide, we were able to detect methyl thiocyanate above cultures of *P. umsongensis* 313P5BS. Conversion of cyanide to thiocyanate is accomplished by bacterial rhodanese and these co-occurring molecules can be extracted simultaneously from headspace above living cultures (Broderick et al., 2008). Weise et al. (2013) have highlighted the importance of bacterial ammonia production and demonstrated accompanied growth inhibition of *Arabidopsis thaliana*. Specific indicator stripes in the TCVA would allow to test for ammonia in the headspace, which would also imply an unspecific inhibition of target organisms.

Some bacteria are well known for pronounced antifungal effects against phytopathogenic fungi. This effect is typical for several strains belonging to *Stenotrophomonas* (Wolf et al., 2002), which was also observed with headspace experiments (Ryan et al., 2009). *Bacillus* species were shown in prior studies not only to produce antifungal VOCs (Fiddaman and Rossall, 1993), but also volatiles that promoted growth in *A. thaliana* (Ryu et al., 2003). Our study demonstrates such antagonistic effects with a robust well plate-based approach and provides various options for modifications to study further effects e.g., growth promotion of bacteria on plants in an adaptable testing system. Moreover, this approach could also be applied to study the prevalence of similar bioactive effects across entire bacterial genera and to correlate volatile effects with the occurrence of strains in particular ecological niches.

As we are convinced that bacterial volatiles might play an important role to modify the composition of host-associated communities, future research needs to focus on the, possibly context-dependent, effects of such small molecules. We anticipate that this newly developed testing approach will be a major step forward to facilitate such studies.

### Conflict of interest statement

The authors declare that the research was conducted in the absence of any commercial or financial relationships that could be construed as a potential conflict of interest.
