# Supplementary material for: A novel assay for the detection of bioactive volatiles evaluated by screening of lichen-associated bacteria
Source: Front Microbiol. 2015 May 1;6:398. doi: 10.3389/fmicb.2015.00398 (PMC4416446; doi:10.3389/fmicb.2015.00398)
Supplement: Supplementary file 1 [file DataSheet1.DOCX]

**Supplementary data**


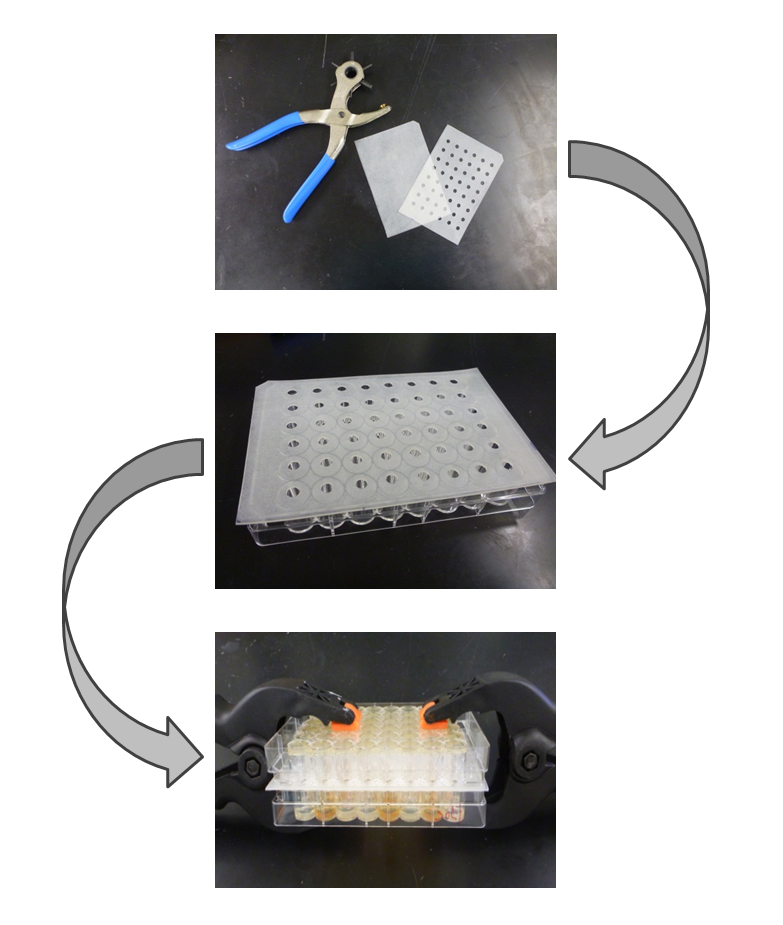


Figure S1: Step by step illustration of a TCVA with two microorganism-containing well-plates. Perforated silicone foil together with two clamps per plate was used to seal adjacent wells and join them into separate chambers. After a microorganism-specific incubation time, the plates were separated again and visually inspected for growth reduction within wells of the target plate.


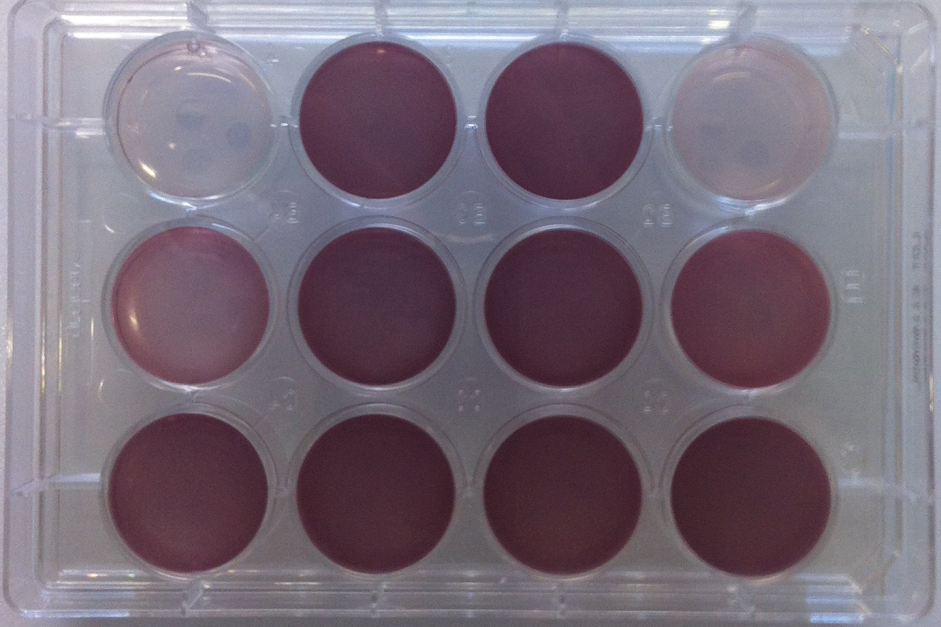


B3

B6

A6

A3

B2

B5

A5

A2

B4

B1

A412

A1

Figure S2: Pretest with *E. coli* K12 and 2–(4–iodophenyl)–3–(4–nitrophenyl)–5–phenyltetrazolium chloride (INT; 0.2 mg/mL) supplemented nutrient agar (NA) 12-well plates. The left plate compartment (A1-A6) was exposed to different Sterilium^TM^ concentrations, while the right plate compartment (B1-B6) was exposed to different Bacilol^TM^ concentrations. High inhibition of *E. coli* was observed with 100 µL Sterilium^TM^ and Bacilol^TM^, respectively (A1 and B1). Lower inhibition was observed with decreasing Sterilium^TM^ and Bacilol^TM^ concentrations (A2-A5 and B2-B5). Two negative controls without any disinfectant were additionally conducted (A6 and B6).


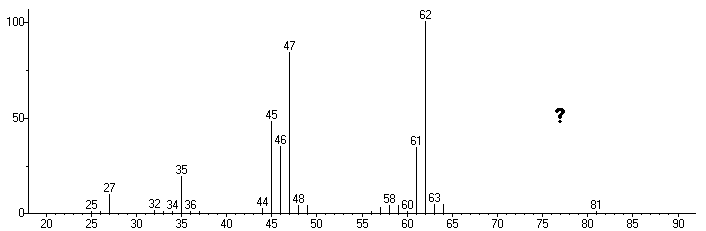


Figure S3: Mass spectrum of unidentified substance (compound ID: 1).


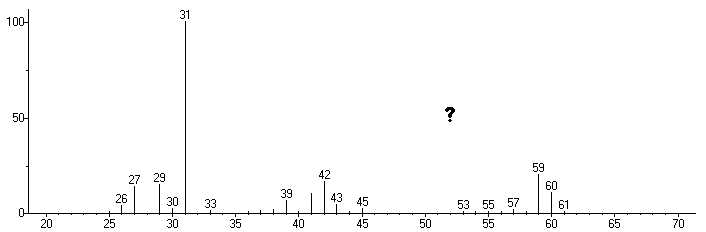


Figure S4: Mass spectrum of unidentified substance (compound ID: 2).


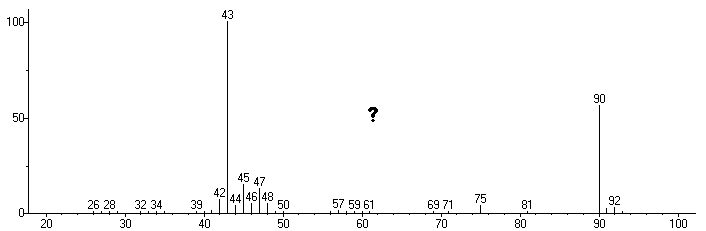


Figure S5: Mass spectrum of unidentified substance (compound ID: 6).


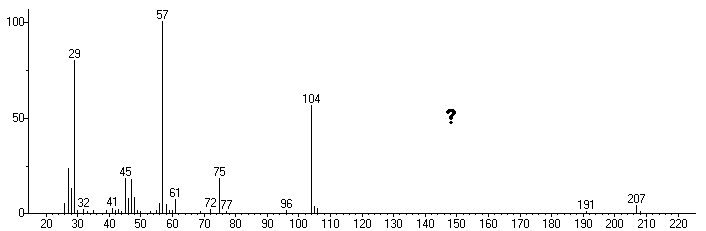


Figure S6: Mass spectrum of unidentified substance (compound ID: 9).


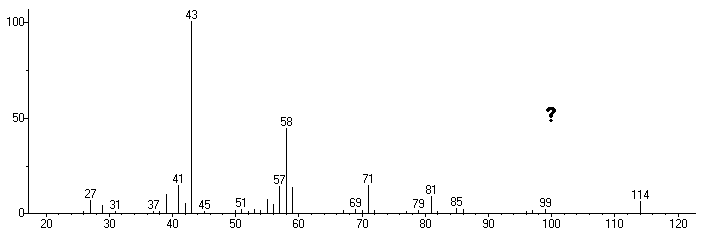


Figure S7: Mass spectrum of unidentified substance (compound ID: 10).


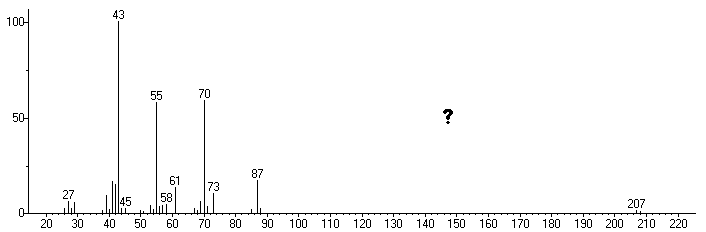


Figure S8: Mass spectrum of unidentified substance (compound ID: 11).


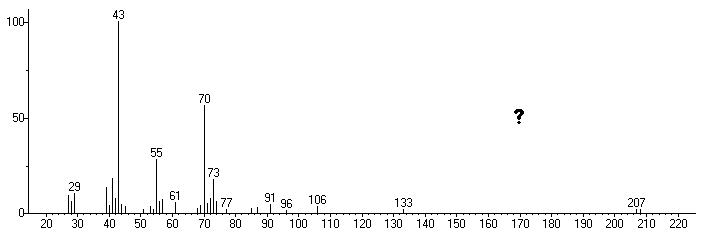


Figure S9: Mass spectrum of unidentified substance (compound ID: 12).


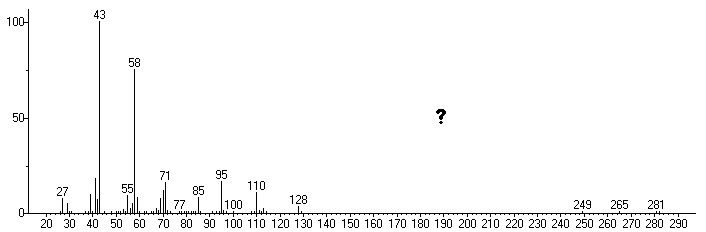


Figure S10: Mass spectrum of unidentified substance (compound ID: 13).


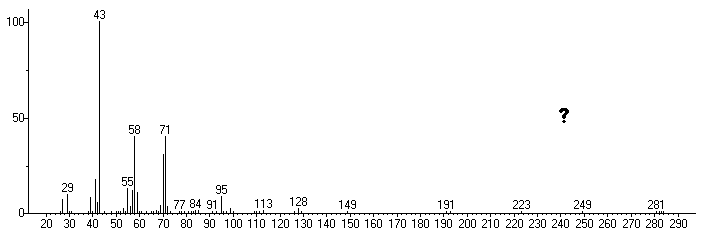


Figure S11: Mass spectrum of unidentified substance (compound ID: 14).


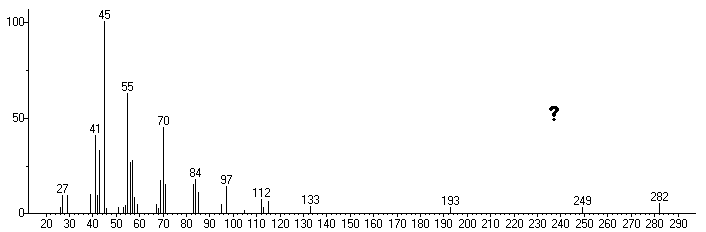


Figure S12: Mass spectrum of unidentified substance (compound ID: 15).


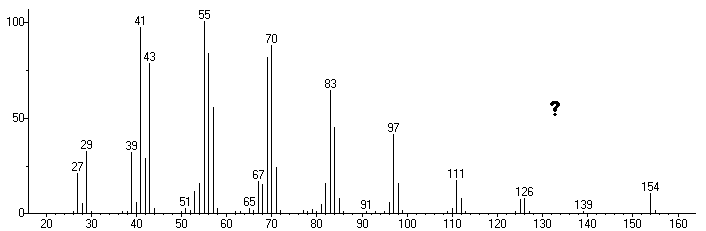
Figure S13: Mass spectrum of unidentified substance (compound ID: 16).


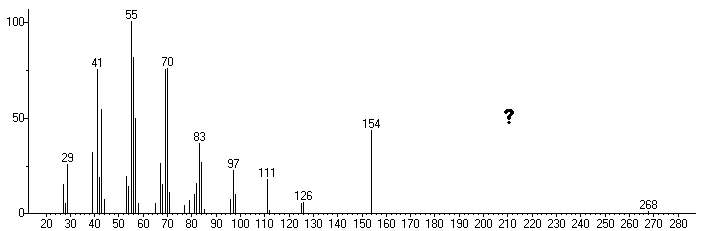


Figure S14: Mass spectrum of unidentified substance (compound ID: 17).


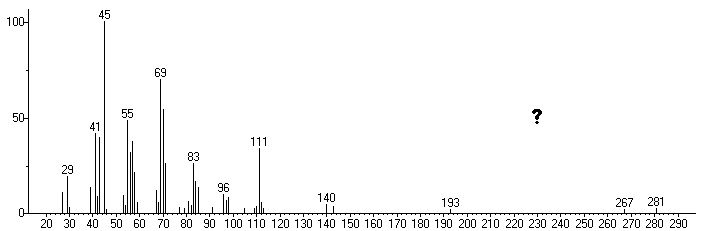


Figure S15: Mass spectrum of unidentified substance (compound ID: 18).


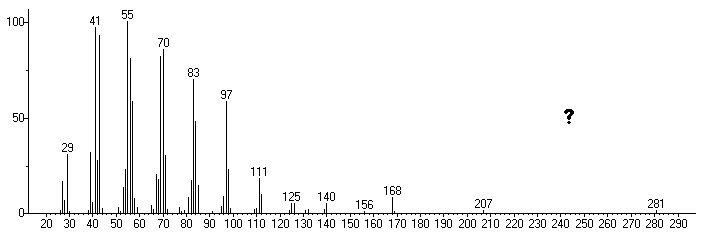


Figure S16: Mass spectrum of unidentified substance (compound ID: 19).


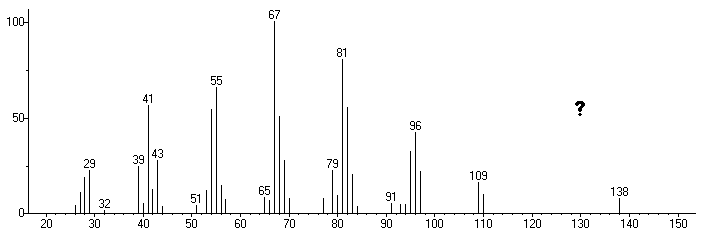


Figure S17: Mass spectrum of unidentified substance (compound ID: 20).


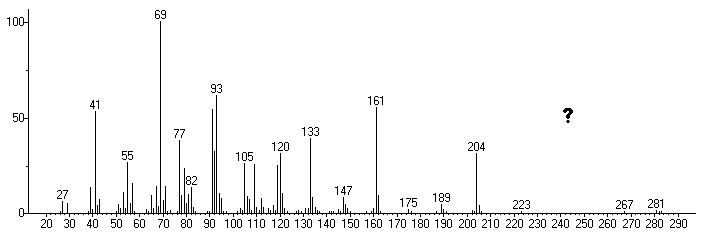


Figure S18: Mass spectrum of unidentified substance (compound ID: 21).

Table S1: The utilized silicone foils were obtained from a local reseller (Hostra GmbH, Graz, Austria). To allow reproducibility of our experiments we have summarized the most important properties.

Table S2: Unique volatile metabolites in the headspace of three bacterial isolates. VOCs from *Bacillus* sp. 43P2BR, *Pseudomonas* sp. 313P5BS and *Stenotrophomonas* sp. 418P4B were detected and specific peaks for each isolate were extracted from chromatogram overlays. Compounds were identified by comparison with reference substance mass spectra and their respective retention indices.
